# Tuberculous Arthritis of the Knee with Rice Body Formation: A Report of a Rare Case

**DOI:** 10.1155/2020/6369781

**Published:** 2020-01-09

**Authors:** Truong Nguyen Khanh Hung, Tran Binh Duong, Tran Phuoc Binh, Dao Thanh Tu, Huynh Phuoc Hau, Truong Trong Tin, Cao Thi, Le Van Tuan

**Affiliations:** ^1^College of Medicine, Taipei Medical University, Taipei City, Taiwan; ^2^Orthopedic and Trauma Department, ChoRay Hospital, Ho Chi Minh City, Vietnam; ^3^Orthopedic and Rehabilitation Department, University of Medicine and Pharmacy at Ho Chi Minh City, Ho Chi Minh City, Vietnam

## Abstract

In this report, we present the case of a 53-year-old man with rice body formation in the right knee caused by tuberculous arthritis (TB arthritis). The patient visited our hospital in January 2018 with a seven-month history of swelling and pain in the right knee. He had no previous history of tuberculosis, and the results of the routine laboratory tests were within normal limits; he also tested negative for rheumatoid factor. Magnetic resonance (MR) imaging revealed multiple rice bodies in the right knee, measuring 5-8 mm. He underwent an arthroscopic operation in the right knee in January 2018 and received antituberculosis polytherapy for 6 months. He was followed-up for more than 01 year. The patient regained good function of the operated knee with no evidence of recurrence during the last follow-up in February 2019. *Conclusion*. The biggest challenge in diagnosing tuberculosis arthritis is the consideration of its possibility in the differential diagnosis, not only in endemic countries where tuberculosis is frequent. A high level of suspicion for TB should be maintained for every infection of the knee joint, particularly in the case of intra-articular rice bodies.

## 1. Introduction

Tuberculosis (TB) is a common and endemic disease in developing countries [1]. Approximately 10.4 million patients develop active infections worldwide, resulting in almost 1.4 million deaths annually [2]. Although the majority of newly diagnosed cases have pulmonary TB, various organs, bones, and joints may also be affected. Skeletal TB in particular accounts for approximately 10% to 35% of extrapulmonary cases, and the knee is the third most frequently affected site after the spine and hip [3]. Rice body formation in the knee is inconsistent but common in tuberculous and rheumatoid arthritis (RA) or/and tuberculous synovitis. However, it is not commonly encountered by arthroscopic surgeons [4]. Intra-articular rice body formation can occur in chronic inflammatory diseases such as RA, TB arthritis, chronic fungal infections, synovial chondromatosis, pigmented villonodular synovitis, gout, or systemic lupus erythematosus (SLE) [4, 5]. We present the case of a patient requiring arthroscopy of the right knee for arthritis, without a history of pulmonary TB or RA.

This review is intended to report a recent clinical case of TB arthritis of the knee and highlight the need for high levels of suspicion to correctly diagnose TB arthritis. The current concepts of clinical and surgical therapies for this condition have also been discussed.

## 2. Case Presentation

A 53-year-old male driver suffering from knee pain was referred to our orthopedic department in January 2018. He provided a history of continuous and progressive right knee pain since the past 1 year without any constitutional symptoms. On physical examination, his knee was swollen with restricted motion (range of movement 0–100 degrees). Pain was elicited on lateral compression of the femoral condyle. The fluid collected twice during knee arthrocentesis was clear.

**Figure 1:**
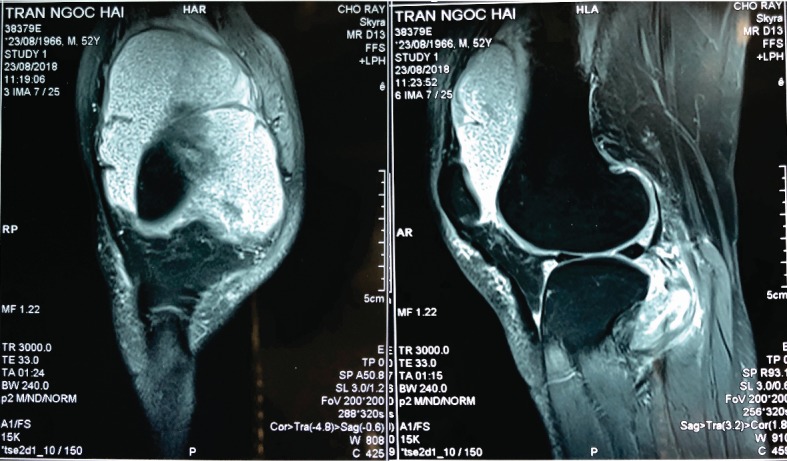
MRI showed multiple rice bodies.

**Figure 2:**
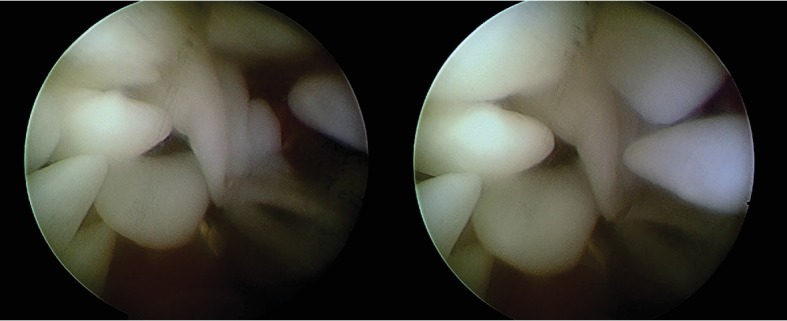
Rice bodies are seen during arthroscopy.

Radiographs of the chest and affected knee appeared normal. He had no previous history of tuberculosis, and the results of the routine laboratory tests were within normal limits; he also tested negative for rheumatoid factor. Examination of the blood revealed a white blood cell (WBC) count of 11.38 G/L, C-reactive protein (CRP) level of 5.0 mg/dL, erythrocyte sedimentation rate (ESR) of V1: 34 mm/h and V2: 71 mm/h, and a rheumatoid factor level of 18.6 IU/mL. MRI showed multiple rice bodies measuring 5-8 mm in both T1- and T2-weighted sequences (Figure 1). The patient underwent arthroscopic surgery (Figure 2).

**Figure 3:**
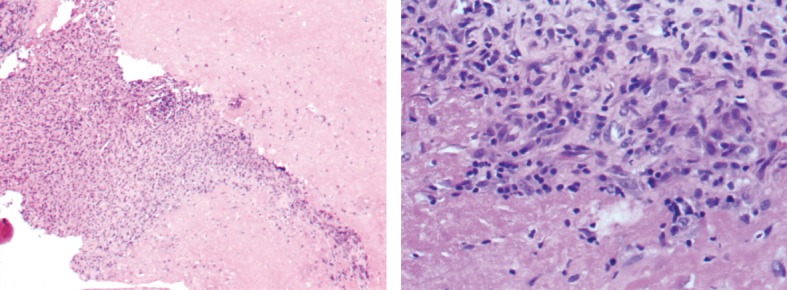
Synovial biopsy result.

The histopathologic exam revealed granulomatous inflammation with caseous necrosis, suggestive of TB (Figure 3). This is the gold standard to diagnose TB. The polymerase chain reaction (PCR) test for *Mycobacterium tuberculosis* DNA was positive.

**Figure 4:**
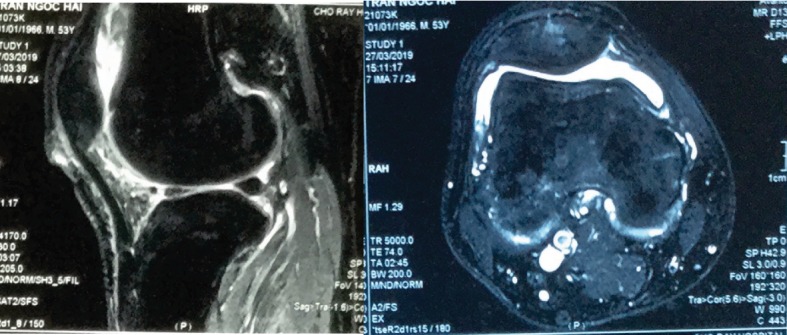
MRI after 3 months.

**Figure 5:**
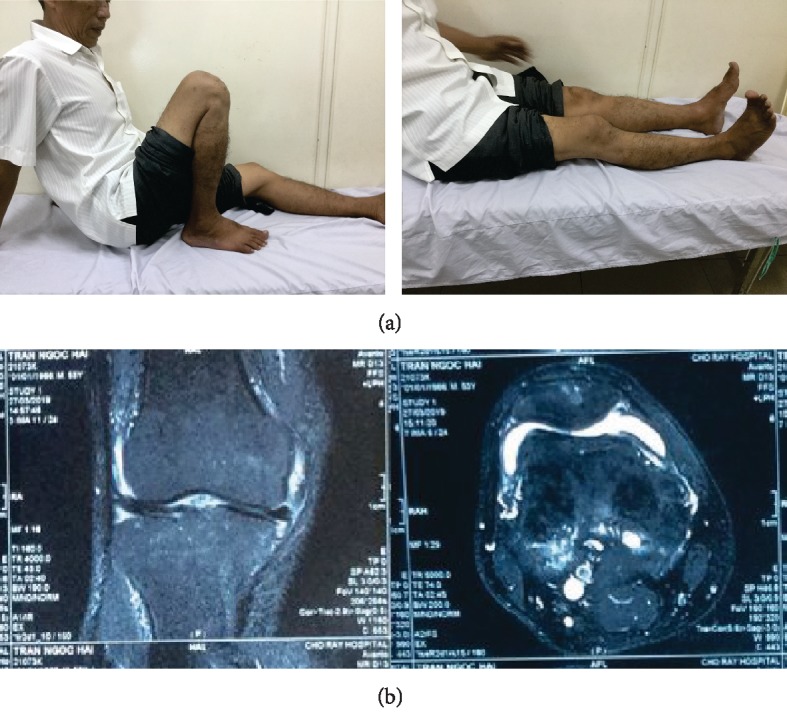
(a) The last follow-up, more than 1 year. (b) MRI of the last follow-up.

The patient was treated with antituberculosis polytherapy that continued for 6 months; it consisted of rifampin, isoniazid, pyrazinamide, and ethambutol in the intensive phase (usually based on 4 drugs) lasting 2 months and isoniazid and ethambutol in the continuation phase (usually with 2 drugs administered) lasting 4 months (2HRZE/4HR). The patient was checked follow up 1 month, 3 months (Figure 4), 6 months and 1 year, respectively. At more than 1-year follow-up, the patient experienced relief from pain and swelling with knee movements ranging from 0 to 120 degrees (Figure 5(a)). The follow-up MRI showed no rice bodies in the T1- and T2-weighted sequences (Figure 5(b)).

The patient provided consent to publish his clinical data in this case report. There are no conflicts of interest to declare.

## 3. Discussion

Rice bodies are shiny white structures with variable sizes and shapes. The shapes may either be like teardrops and nodules or be angular and flake-like [6]. In 1980, Cheung et al. elucidated a theory of synovial origin for rice body formation, in which the synovium undergoes inflammation followed by tiny infarcts and peeling of the infarcted tissue [7]. Two years later, Popert et al. proposed a theory of origin in the synovial fluid, independent of the synovial elements [8]. Musculoskeletal TB may manifest without constitutional symptoms. Pulmonary involvement has been found in only one-third of musculoskeletal TB cases leading to delays in diagnosis [9]; laboratory parameters such as ESR do not have much diagnostic value [10]. Drew et al. suggested that the diagnosis of TB arthritis requires a thorough history emphasizing on relevant travel, a careful physical examination, and appropriate diagnostic testing. In their report, the time for delayed diagnosis was between 2 months and 10 years [11].

According to the global tuberculosis (TB) report, 2017, of the World Health Organization, Vietnam is one of the high-burden countries with 13593 cases of diagnosed TB in 2016 [12]. However, musculoskeletal TB may manifest without constitutional symptoms. It is therefore essential to consider TB in the differential diagnosis as it may easily be misdiagnosed, particularly in patients without a history of TB or specific symptoms. TB should be considered a possible offending infectious agent in cases of unexplained joint pain and swelling. Arthroscopy with antituberculosis polytherapy is an effective treatment, but more research and a long-term follow-up process are necessary to observe the patient effectively.

According to all the reports, the gold standard for diagnosing TB arthritis is synovial biopsy, with positive results in 80% of cases [13, 14]. The findings typical of TB arthritis include granulomas, lymphocytes, and giant cells with caseation. Treatment may involve the use of splints for a short time to decrease acute and chronic symptoms in specific cases of TB arthritis and to prevent deformation of infected joints [15–17]. Surgical procedures should be limited to joints with severe cartilage destruction, deformation of infected joints, large abscesses, and infections with multidrug-resistant or atypical mycobacteria [16, 18]. The main treatment is antituberculosis polytherapy (for 12-18 months) and active-assisted non-weight-bearing exercises of the relevant joints throughout the postoperative period. Operative interventions (synovectomy and debridement) are required when the patient is not responding after 4-5 months of antituberculosis polytherapy [19, 20].

## 4. Conclusion

TB is still an important global public health problem. TB arthritis accounts for approximately 1-3% of all cases of TB and 10-11% of extrapulmonary TB cases [20]. The biggest challenge in diagnosing tuberculosis arthritis is to consider the possibility in the differential diagnosis, not only in endemic countries where tuberculosis is frequent. A high level of suspicion should be maintained for TB in every infection of the knee joint, particularly in cases with intra-articular rice bodies [13, 14, 20]. An early diagnosis and specific and adequate treatment may improve the possibility of maintaining good joint function [15–17]. The main treatment is antituberculous polytherapy (for 12-18 months) and active-assisted non-weight-bearing exercises of the relevant joint throughout the postoperative period [19, 20]. A high level of suspicion for TB should be maintained for every infection of the knee joint, particularly in the case of intra-articular rice bodies.

## Supplementary Materials

Supplementary Materials During arthroscopy, we also see so many intra-articular rice bodies, with hundreds of white coloured shiny round to oval bodies with size ranging from 5 to 8 millimetres. After washing out the remaining rice bodies, arthroscopic synovectomy was performed for synovial biopsy.
